# Shape Memory Alloys Applied to Automotive Adaptive Aerodynamics

**DOI:** 10.3390/ma16134832

**Published:** 2023-07-05

**Authors:** Miriam Battaglia, Andrea Sellitto, Angela Giamundo, Michele Visone, Aniello Riccio

**Affiliations:** 1 Department of Engineering, University of Campania “Luigi Vanvitelli”, Via Roma 29, 81031 Aversa, Italy; andrea.sellitto@unicampania.it (A.S.); aniello.riccio@unicampania.it (A.R.); 2 BLUE Engineering S.r.l., Via Ex Aeroporto 30/32, 80038 Pomigliano d’Arco, Italy; a.giamundo@blue-group.it (A.G.); m.visone@blue-group.it (M.V.)

**Keywords:** shape memory alloys, adaptive aerodynamics, actuators, morphing, FEM

## Abstract

Shape memory alloys (SMAs) are gaining popularity in the fields of automotive and aerospace engineering due to their unique thermomechanical properties. This paper proposes a numerical implementation of a comprehensive constitutive model for simulating the thermomechanical behavior of shape memory alloys, with temperature and strain as control variables to adjust the shape memory effect and super elasticity effect of the material. By implementing this model as a user subroutine in the FE code Abaqus/Standard, it becomes possible to account for variations in material properties in complex components made of shape memory alloys. To demonstrate the potential of the proposed model, a skid plate system design is presented. The system uses bistable actuators with shape memory alloy springs to trigger plate movement. The kinematics and dynamics of the system are simulated, and effective loads are generated by the shape memory alloy state change due to the real temperature distribution in the material, which depends on the springs’ geometrical parameters. Finally, the performance of the actuator in switching between different configurations and maintaining stability in a specific configuration is assessed. The study highlights the promising potential of shape memory alloys in engineering applications and demonstrates the ability to use them in complex systems with accurate simulations.

## 1. Introduction

Shape memory alloys (SMA) are a group of metal materials that can return to their original shape after being deformed by exposure to a specific input such as mechanical load, heat, or a magnetic field [1]. These alloys have two distinct crystal structures called the austenitic and martensitic phases. The transformation between these phases is responsible for the shape memory effect and is reversible under certain conditions. SMAs have a wide range of applications in various fields, including aerospace, biomedical engineering, robotics, and smart materials [2,3,4,5,6,7]. 

Shape memory materials are characterized by the shape memory effect (SME), super elasticity (SE), also called pseudoelasticity (PE), high damping capabilities, and adaptive properties related to the reversibility of phases’ transition. Nowadays, shape memory alloys are the most widely explored shape memory materials [8,9,10,11].

The shape memory effect (SME) is a unique property of these materials that allows them to remember their original shape even after undergoing several deformations. The changes in shape that occur due to temperature variations are primarily attributed to the transformation of the martensitic phase. The SME is a phenomenon wherein the material can recover its original shape, even if it is deformed at low temperatures. The superelastic effect (SE) is characterized by the recovery of deformation after unloading. This behavior is observed during loading and unloading at high temperatures and is attributed to stress-induced martensitic transformation. 

The mechanical behavior of shape memory alloys is a consequence of the transition between two distinct phases that possess specific crystal structures, namely the austenite and the martensite phases. The austenite phase (A), which is stable at high temperatures, possesses a symmetric structure. Conversely, the martensite phase (M), which is stable at low temperatures, possesses a generally non-symmetric structure. The shift from the Austenite to the martensite phase is induced by a decrease in material temperature or an increase in stress and can be considered a solid–solid non-diffusive transformation [12,13]. Ni-Ti austenite has a cubic structure with relatively high entropy, leading to energy dissatisfaction. As the austenite is cooled, there is a significant reduction in Young’s modulus. With further cooling, the austenitic phase transforms into two alternative phases with lower entropy. These phases include a B19′ monoclinic martensite and a distorted rhombic martensite (the R phase). Both are dependent on thermodynamic and kinetic conditions for their formation. The martensite phase is more thermally stable than the R phase and is, therefore, preferred. The R phase forms earlier due to its low activation energy, giving it a kinetic advantage [14]. Moreover, the martensite crystal structure can be found in either twinned form (stress-induced transformation) or detwinned form (temperature-induced transformation).

Numerous studies have been conducted to investigate the effects of thermomechanical treatments on the unique properties of shape memory materials, as they are highly sensitive to external loads. Assawakawintip et al. found that heat treatments have a greater impact on alloys than time [15]. Their research on shape memory alloy (SMA) springs revealed that curing processes at high temperatures (500 °C) significantly enhanced the transition plateau slope and strength, and reduced hysteresis. Additionally, aging heat treatments were found to increase transition temperatures. Notably, post-heat treatments were observed to have a more significant influence on the mechanical properties of the materials compared to the composition of the alloys themselves [16]. Moreover, a study conducted by Tsaturyants et al. [17] found that subjecting a material to 10 or more thermal cycles can significantly enhance its mechanical properties. When a thermal process involving 100 heating–cooling cycles is applied, an increase in the maximum stress and yield stress of the dislocations is observed, as well as an improvement in the recovery strain of about 1 percent. Indeed, the temperatures of inverse martensitic transformations are closely linked to both the grain/subgrain size and the presence of Ti3Ni4 precipitates [18]. The impact of heat treatment on the evolution of Ti3Ni4 precipitates was investigated by Ren et al. [19] to determine the critical size required for total dissolution within the matrix.

In the literature, there are several analytical constitutive models that can describe the thermomechanical behavior of SMA materials. These models can be divided into three categories: chemical models that simulate changes in the crystal structure, analytical models that adopt ad hoc thermomechanical relations and micromechanics-based models. Some chemical models highlight substantial differences in kinetic transformations between phases, describing mono-dimensional laws of SMA materials [20]. Other authors have adopted three-dimensional approaches [21,22,23] that allow for a more accurate description of phenomenology. However, due to the lack of multiaxial experimental data, this approach is difficult to validate. On the other hand, thermodynamic models are based on experimental evidence and involve the definition of laws that can describe the variation of thermal and mechanical parameters during SMA transformation. These models are relatively simple to implement in numerical applications and are widely used for practical purposes [24]. Some thermodynamic models adopt multidimensional constituent laws based on the variation of the martensite volumetric fraction [25,26]. However, more recent works have presented new advanced models that are able to describe the onset and evolution of SMA transformation under multiaxial loading conditions [27]. Finally, micromechanical models allow for the determination of the material’s macroscopic behavior by starting from a detailed description of the crystallographic structure and microscopic deformation mechanisms. These models consider the martensite volumetric variation as an additional transformation and use micromechanics to evaluate the global energy involved in the phase transformation. The transformation is assessed in a control volume, and the stress/strain distributions are evaluated as volumetric averaged variations [28,29,30,31,32,33]. While micromechanic-based constitutive models are the most representative of the physical phenomena behind the phase transformation of SMAs, they are also the most complex to implement in simulation tools due to the high number of required input parameters and the very high computational cost involved.

In the last few decades, SMAs have been adopted for numerous industrial applications, ranging from automotive to aerospace engineering. SMA wires have been used as actuators [34,35,36] or embedded in laminated composites to drive surface morphing [37,38,39]. However, the literature suggests that several difficulties have been encountered in these applications, mainly related to the fracture mechanisms at the interface between SMAs and composites, as well as the need to maintain SMA temperatures within specific ranges for the stable activation of both the actuators and the morphing surfaces [40,41,42]. Furthermore, to overcome the limitations of constant force and cooling processes associated with shape memory alloys, some studies have explored different approaches to utilize the benefits of the austenite and martensite phase transformation [43]. 

The primary aim of this study is to propose and assess the feasibility of utilizing shape memory alloys for the movement of a skid plate using a bistable SMA actuator and evaluate its aerodynamic advantages. The thermodynamic constitutive model of the material was incorporated as a UserMAT in the ABAQUS finite element code to enable numerical simulation of both characteristic effects of these alloys simultaneously. Incorporating both primary effects in the numerical model is crucial for using shape memory alloys as actuators. Moreover, using a user material subroutine (UMAT), different states within the spring for both the pseudoelastic effect and the shape memory effect could be considered during the simulation, resulting in a highly realistic representation of the physical phenomenon. Thus, this approach offers significant advantages in terms of simulation, design, support for experimental tests, and understanding of the physical phenomenon. Although physical modeling of the SMA would be useful to gain a deeper understanding, the focus was on developing a tractable mathematical model suitable for engineering applications.

In Section 2, the thermodynamic constitutive model for SMAs is introduced. In Section 3, a description of the constitutive model implementation in the ABAQUS user material subroutine is provided. In Section 4, the application to the design of the bistable actuator for the skid plate system is introduced and discussed. Conclusions are reported in Section 5.

## 2. Theoretical Background

In this section, the 3D phenomenological constitutive model of shape memory alloy is presented. The thermodynamic model details the behavior of shape memory alloys under the action of varying thermal and mechanical loads. The thermal and mechanical behavior is completely described by coupling two laws: the thermomechanical and kinetic laws [44,45,46]. The Brinson model has been chosen as the fundamental framework for investigating the behavior of shape memory alloys in this study.

### 2.1. Thermomechanical Law

The thermomechanical law provides a relationship between global elastoplastic deformation and elastic and transformational deformation. In this work, classical hyperplastic velocity theory [46,47,48] was adopted to model this relationship. According to this theory, the total deformation rate can be seen as the sum of an elastic (${\dot{\varepsilon }}_{el}$) and a transformation related (${\dot{\varepsilon }}_{tr}$) contribution:

(1) $$\dot{\varepsilon }=\dot{\varepsilon_{el}}+\dot{\varepsilon_{tr}}$$

The stress–strain relationship has been evaluated by the Jaumann rate of Cauchy stress, known as the co-rotational stress rate [49], and can be expressed by the thermomechanical law given in Equation (2):

(2) $$\dot{\sigma }=R\dot{\varepsilon_{el}}+k\dot{T}=R\left(\dot{\varepsilon }-\dot{\varepsilon_{tr}}\right)+k\dot{T}$$

where *R* is the elastic tensor, *k* is the thermal modulus tensor, and *T* is the current temperature.

The phase transition from austenite to martensite leads to the formation of twinned martensite or detwinned martensite. The difference between the two consists of the orientation of the atoms in the crystalline structure [25] and the formation process.

Equation (3) defines a relationship between the evolution of the transformation deformation and the volumetric fraction of martensite during the transformation from austenite to martensite and vice versa. According to the Jaumann rate of Cauchy stress, the strain rate related to the phase transformation can be expressed in terms of martensite volume fraction rate ($\dot{\xi }$) according to Equations (3).

(3) $${\dot{\varepsilon }}_{tr}=\dot{\xi }E$$

where the transformation tensor *E* depends on the phase transformation (from martensite to austenite or vice versa) as shown in Equation (4), and $\dot{\xi }$, as mentioned earlier, is representative of the evolution of martensite volumetric fraction.

(4) $$E=\left\{\begin{array}{c} \frac{2}{3}\begin{array}{cc} \varepsilon_{tr}^{max}\frac{\sigma ’}{\overset{-}{\sigma ’}} & when\;\dot{\xi }>0 \end{array}\\ \begin{array}{cc} \varepsilon_{tr}^{max}\frac{\varepsilon_{tr}}{\left\|\varepsilon_{tr}\right\|} & when\;\dot{\xi }<0 \end{array} \end{array}\right.$$

In Equation (4), the volumetric fraction rate of martensite is positive when passing from austenite to martensite. The volumetric fraction rate of martensite is negative when passing from martensite to austenite [50].

### 2.2. Kinetic Law

The kinetic law is adopted to determine the state of the shape material alloy as a function of its initial conditions for applied strains and temperature. This methodology allows us to evaluate the variation of the volumetric fraction of martensite.

**Figure 1:**
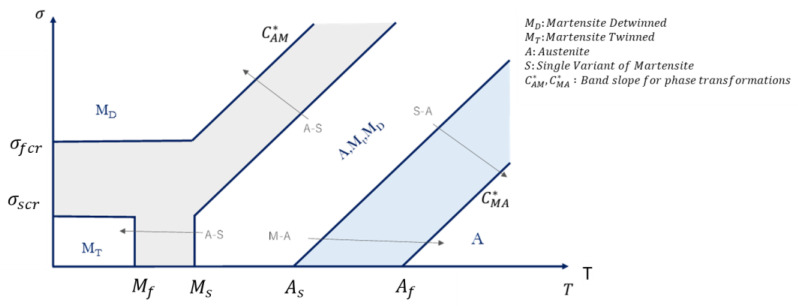
Phase diagram of a shape memory alloy (*σ*-T diagram phase [44]).

**Figure 2:**
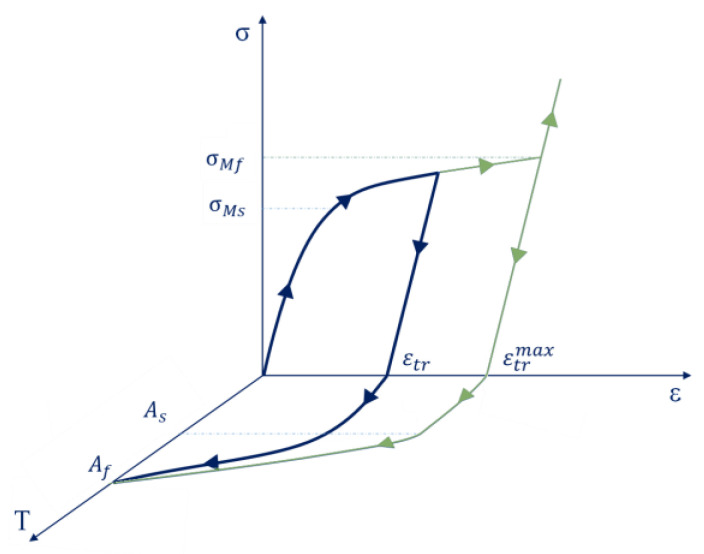
Shape memory effect.

According to the phase diagram (Figure 1), the following parameters can be defined [51,52]:

(5) $$C_{AM}^{*}=\frac{C_{AM}}{E_{M}}$$

(6) $$C_{MA}^{*}=\frac{C_{MA}}{E_{A}}$$

(7) $$\varepsilon_{scr}=\frac{\sigma_{scr}}{E_{A}}$$

(8) $$\varepsilon_{fcr}=\frac{\sigma_{fcr}}{E_{M}}+\varepsilon_{tr}^{max}$$

where, $C_{AM}^{*}$ and $C_{MA}^{*}$ are the band slope for forward and reverse phase transformations in the (*σ*, *T*) diagram, while *C_AM_* and *C_MA_* are the band slope for forward and reverse phase transformations in the (*ε*, *T*) diagram. *E_A_* and *E_M_* are Young’s modulus of the austenite and martensite phases, while $\sigma_{scr}$ and $\sigma_{fcr}$ are the austenite to martensite critical stress values for the start and end of the phase transformation. Figure 2 shows the constitutive relation of a shape material alloy for a mono-dimensional state. To represent three-dimensional stress states, equivalent stresses and equivalent strains have to be considered.

The concept of the shape memory effect is illustrated in Figure 2. During the loading and unloading phase in a condition of low temperature, residual deformation ($\varepsilon_{tr}$) can be appreciated in the shape material alloy. This residual deformation (which can reach a maximum value of $\varepsilon_{tr}^{max}$) depends on the maximum stress induced by the load. The green curve represents the maximum value of stress that the alloy can withstand. *A_s_* is an additional effect, and the application of a thermal load can reduce strains in shape memory alloys (up to zero for a temperature of *A_s_*).

The previously shown diagrams have been completely characterized for the two basic phase transformations: austenite-to-martensite and martensite-to-austenite phase transformations in the next sub-sections.

### 2.3. Austenite–Martensite Phase Transformation

As shown in Figure 1, the state of the shape memory alloy is defined by stress and temperature conditions. Considering a starting reference temperature *T*_0_, the variations of strain and stress during the transition from the austenite to martensite phase (grey zone in Figure 1) can be evaluated according to Equations (9) and (10).

(9) $$\varepsilon_{Ms}=\left\{\begin{array}{cc} C_{AM}^{*}\left(T-T_{0}\right)+\varepsilon_{eq} & if\;T_{0}\geq M_{s}\\ \varepsilon_{eq} & if\;T_{0}\leq M_{s} \end{array}\right.$$

(10) $$\sigma_{Ms}=\left\{\begin{array}{cc} C_{AM}\left(T-T_{0}\right)+\sigma_{eq} & if\;T_{0}\geq M_{s}\\ \sigma_{eq} & if\;T_{0}<M_{s} \end{array}\right.$$

The threshold values for the start and the end of the phase transformation in terms of strain and stress are estimated according to, respectively, relations (11), (12), (13), and (14).

Transformation start threshold:

(11) $$\varepsilon_{Ms}=\left\{\begin{array}{cc} \varepsilon_{scr}+C_{AM}^{*}\left(T-M_{s}\right) & if\;T_{0}\geq M_{s}\\ \varepsilon_{scr} & if\;T_{0}\leq M_{s} \end{array}\right.$$

(12) $$\sigma_{Ms}=\left\{\begin{array}{cc} \sigma_{cr}+C_{AM}\left(T-M_{s}\right) & if\;T_{0}\geq M_{s}\\ \sigma_{scr} & if\;T_{0}<M_{s} \end{array}\right.$$

Transformation end threshold:

(13) $$\varepsilon_{Mf}=\left\{\begin{array}{cc} \varepsilon_{fcr}+C_{AM}^{*}\left(T-M_{s}\right) & if\;T_{0}\geq M_{s}\\ \varepsilon_{fcr} & if\;T_{0}\leq M_{s} \end{array}\right.$$

(14) $$\sigma_{Mf}=\left\{\begin{array}{cc} \sigma_{fcr}+C_{AM}\left(T-M_{s}\right) & if\;T_{0}\geq M_{s}\\ \sigma_{fcr} & if\;T_{0}<M_{s} \end{array}\right.$$

Referring to the austenite–martensite transformation, the volume fraction rate of martensite is determined by Equation (15).

(15) $$\dot{\xi }=\left(1-\xi \right)\frac{{\dot{\varepsilon }}_{Ms}}{{\varepsilon_{Mf}}^{-\varepsilon }}$$

### 2.4. Martensite–Austenite Phase Transformation

Considering a starting reference temperature *T*_0_, the variations of strain and stress during the transition from martensite to austenite phase (charming zone in Figure 1) can be evaluated according to Equations (16) and (17).

(16) $$\varepsilon_{As}=C_{MA}^{*}\left({T-T}_{0}\right)+\varepsilon_{eq}$$

(17) $$\sigma_{As}=C_{MA}\left(T-T_{0}\right)+\sigma_{eq}$$

Equations (18)–(19) and (20)–(21) evaluate, respectively, the limit values for the starting and ending of the martensite–austenite transformation.

Starting transformation threshold:

(18) $$\varepsilon_{As}=C_{MA}^{*}\left(T-A_{s}\right)+\varepsilon_{tr}^{max}$$

(19) $$\sigma_{As}=C_{MA}\left(T-A_{s}\right)$$

End transformation threshold:

(20) $$\varepsilon_{Af}=C_{MA}^{*}\left(T-A_{f}\right)$$

(21) $$\sigma_{Af}=C_{MA}\left(T-A_{f}\right)$$

For the martensite–austenite transformation, the volume fraction rate of martensite is determined by Equation (22).

(22) $$\dot{\xi }=\left(1-\xi \right)\frac{{\dot{\varepsilon }}_{As}}{{\varepsilon_{Af}}^{-\varepsilon }}$$

## 3. User Material Subroutine (SMA Constitutive Relations)

Although the behavior of shape memory alloys has been implemented by the finite element method [53], the main effects that characterize shape memory alloys (the shape memory effect and the pseudoelastic effect) are not simultaneously available in the material models commonly adopted by commercial finite element platforms. To overcome this issue, a user material model has been developed to assess the thermomechanical behavior of SMA components at the same time.

The three-dimensional thermomechanical behavior of SMA materials has been implemented in a User MATerial (UMAT) routine for the Abaqus commercial FE code. This UMAT routine allows us to evaluate the crystallographic phase and the stress and the strain conditions related to a thermomechanically applied load. The routine can be split into three sequential macroblocks:

The first block allows us to evaluate the shape memory alloy stiffness matrix starting from the compliance matrix of the two individual material phases. Relations (23) and (24) show the basic compliance matrix for the full austenite and full martensite phases.

(23) $$\left[S_{A}\right]=\left[\begin{array}{c} \begin{array}{cccccc} \frac{1}{E_{A}} & -\frac{\nu_{A}}{E_{A}} & -\frac{\nu_{A}}{E_{A}} & 0 & 0 & 0\\ -\frac{\nu_{A}}{E_{A}} & \frac{1}{E_{A}} & -\frac{\nu_{A}}{E_{A}} & 0 & 0 & 0\\ -\frac{\nu_{A}}{E_{A}} & -\frac{\nu_{A}}{E_{A}} & \frac{1}{E_{A}} & 0 & 0 & 0\\ 0 & 0 & 0 & \frac{1}{G_{A}} & 0 & 0\\ 0 & 0 & 0 & 0 & \frac{1}{G_{A}} & 0\\ 0 & 0 & 0 & 0 & 0 & \frac{1}{G_{A}} \end{array} \end{array}\right]$$

(24) $$\left[S_{M}\right]=\left[\begin{array}{c} \begin{array}{cccccc} \frac{1}{E_{M}} & -\frac{\nu_{M}}{E_{M}} & -\frac{\nu_{M}}{E_{M}} & 0 & 0 & 0\\ -\frac{\nu_{M}}{E_{M}} & \frac{1}{E_{M}} & -\frac{\nu_{M}}{E_{M}} & 0 & 0 & 0\\ -\frac{\nu_{M}}{E_{M}} & -\frac{\nu_{M}}{E_{M}} & \frac{1}{E_{M}} & 0 & 0 & 0\\ 0 & 0 & 0 & \frac{1}{G_{M}} & 0 & 0\\ 0 & 0 & 0 & 0 & \frac{1}{G_{M}} & 0\\ 0 & 0 & 0 & 0 & 0 & \frac{1}{G_{M}} \end{array} \end{array}\right]$$

Values *E_A_*, *E_M_*, *v_A_*, *v_M_*, *G_A_,* and *G_M_* represent, respectively, the elastic modulus, Poisson’s ratio, and the shear modulus of the austenite and martensite phases. 

Once the compliance matrix for the individual phases is individually evaluated (according to the relations above), thanks to the volumetric fraction of martensite (*ξ*), the material’s compliance matrix, at a specific phase transformation state, can be evaluated during the transformation phase as:

(25) $$\left[S\right]=\xi \left[S_{M}\right]+\left(1-\xi \right)\left[S_{A}\right]$$

**Figure 3:**
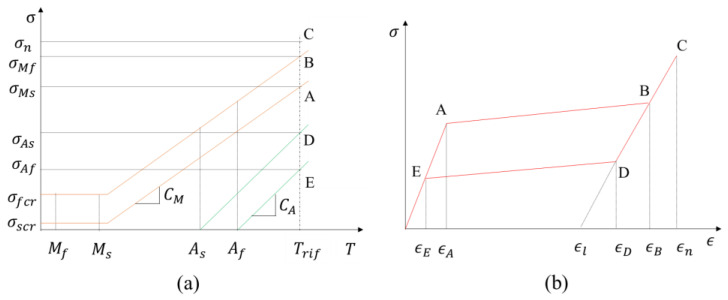
Pseudoelastic effect. (**a**) Tension vs. temperature relationship (orange lines for martensite phase and green lines for austenite phase); (**b**) Tension vs. deformation relationship.

The second block of the material subroutine evaluates the stress–temperature (Figure 3a) and stress–strain (Figure 3b) relationships for each finite element at a specific time/load step. The two diagrams highlight some characteristic points for the austenite to martensite phase transformation and vice versa.

In Figure 3a, the four characteristic temperatures of martensitic transformation are depicted: *M_f_* (martensite finish transformation stress), *M_s_* (martensite start transformation stress), *A_s_* (austenite start transformation stress), and *A_f_* (austenite finish transformation stress). In shape memory alloys (SMA), Direct Martensite Transformation (DMT) and Reverse Martensite Transformation (RMT) are crucial processes that impact the shape memory properties of the material. The DMT involves cooling the SMA alloy below the start temperature of martensitic transformation (*M_s_*), leading to the formation of a martensitic structure that can be used to temporarily store a shape. This process is reversible. Conversely, during the RMT, the SMA alloy is heated above the onset temperature of martensitic transformation (*M_s_*), causing the martensitic structure to transform back into austenite. According to a study by Santamarta et al., RMT temperatures are higher than DMT temperatures. This process is also reversible and can be used to restore the original shape of the SMA alloy. For the current research, only the RMT temperature was considered. The ability of the SMA alloy to change and recover its shape with the application of a thermal heating load was developed as part of the numerical procedure.

With reference to Figure 3a, the *A*, *B*, *D*, and *E* locations are characterized, for a given temperature T*, respectively, by the following values of the stress: $\sigma_{M}^{S}$ martensite starts transformation stress, $\sigma_{M}^{f}$ martensite finish transformation stress, $\sigma_{A}^{S}$ austenite starts transformation stress, and $\sigma_{A}^{f}$ austenite finish transformation stress. 

These stresses are evaluated according to the following relations:

(26) $$\sigma_{M}^{s}=\sigma_{scr}+C_{M}\left(T^{*}-M_{s}\right)$$

(27) $$\sigma_{M}^{f}=\sigma_{fcr}+C_{M}\left(T^{*}-M_{s}\right)$$

(28) $$\sigma_{A}^{s}=C_{A}\left(T^{*}-A_{s}\right)$$

(29) $$\sigma_{A}^{f}=C_{A}\left(T^{*}-A_{f}\right)$$

In stress–strain diagram, shown in Figure 3b, the strain values at *A*, *B*, *D*, and *E* locations can be evaluated by considering the elastic properties of the austenite and martensite phases, according to the following relations:

(30) $$\varepsilon_{A}=\frac{\sigma_{M}^{s}}{E_{A}}$$

(31) $$\varepsilon_{B}=\frac{\sigma_{M}^{f}}{E_{M}}+\varepsilon_{l}$$

(32) $$\varepsilon_{D}=\frac{\sigma_{A}^{s}}{E_{M}}+\varepsilon_{l}$$

(33) $$\varepsilon_{E}=\frac{\sigma_{A}^{f}}{E_{A}}$$

The third block of the material routine evaluates the stress thermal matrices of the full austenite and martensite phases, according to the following relations:

(34) $$\left[K_{A}\right]=\left[\begin{array}{cccccc} \frac{\alpha_{A}E_{A}}{1-2\nu_{A}} & 0 & 0 & 0 & 0 & 0\\ 0 & \frac{\alpha_{A}E_{A}}{1-2\nu_{A}} & 0 & 0 & 0 & 0\\ 0 & 0 & \frac{\alpha_{A}E_{A}}{1-2\nu_{A}} & 0 & 0 & 0\\ 0 & 0 & 0 & 0 & 0 & 0\\ 0 & 0 & 0 & 0 & 0 & 0\\ 0 & 0 & 0 & 0 & 0 & 0 \end{array}\right]$$

(35) $$\left[K_{M}\right]=\left[\begin{array}{cccccc} \frac{\alpha_{M}E_{M}}{1-2\nu_{M}} & 0 & 0 & 0 & 0 & 0\\ 0 & \frac{\alpha_{M}E_{M}}{1-2\nu_{M}} & 0 & 0 & 0 & 0\\ 0 & 0 & \frac{\alpha_{M}E_{M}}{1-2\nu_{M}} & 0 & 0 & 0\\ 0 & 0 & 0 & 0 & 0 & 0\\ 0 & 0 & 0 & 0 & 0 & 0\\ 0 & 0 & 0 & 0 & 0 & 0 \end{array}\right]$$

where *α_A_* and *α_M_* are the thermal coefficients for the austenite and martensite phases respectively. Then, the stress thermal matrix [*K_T_*] for the phase transition state is evaluated to update the stress tensor. The thermal matrix [*K_T_*] is evaluated according to the following equation:

(36) $$\left[K_{T}\right]=\xi \left[K_{M}\right]+\left(1-\xi \right)\left[K_{A}\right]$$

To update the stress tensor, the following relation is adopted:

(37) $$\sigma =\sigma_{0}+d\sigma$$

where *σ*_0_ is the stress tensor at the previous step, and the stress tensor increment *dσ* can be expressed as:

(38) $$\left[d\sigma \right]=\left[{d\sigma }_{M}\right]+\left[K_{T}\right]\left(T_{1}-T^{*}\right)+\left[dK_{T}\right]\left(T_{1}-T_{0}\right)$$

${d\sigma }_{M}$ is the stress referred only to the mechanical behavior, *T*_1_ is the current temperature, and *T*_0_ is the temperature in the previous step.

The thermal matrix increment is defined as (Equation (39)):

(39) $$\left[{dK}_{T}\right]=d\xi \left(\left[K_{M}\right]-\left[K_{A}\right]\right)$$

where dξ is the martensite volume fraction variation.

## 4. Bistable SMA Actuator for a Skid Plate

Shape memory alloys (SMA) are part of the smart materials group. The major quality characteristics of SMAs are the shape memory effect (SME) and superelasticity (SE). Due to these two main effects, the material is able to mechanically deform and maintain the deformed shape until a thermal load is applied. These capabilities make them particularly suitable as actuators.

In the frame of preliminary design analysis, the present study investigates the behavior of a bistable actuator based on shape memory alloys that are characterized by two springs operating in opposition of the phase. At the reference temperature, the SMA springs are subjected to a compressive load to initiate spring transformation in the martensitic phase. However, after removing the mechanical load, the material can only recover a small portion of the residual deformation (3%), which is significantly less than the applied mechanical load. Full recovery is achieved only when thermal loading is applied to trigger the transition from the martensitic to the austenitic phase.

Bistable actuators are characterized by a key feature that addresses a major challenge in SMA actuators. The ability of these actuators to switch between configurations and maintain stability without the requirement of keeping the SMA elements consistently above the activation threshold temperature has been utilized in a skid plate system. The designed actuator can switch from one configuration to another by heating one or the other spring and maintain the balanced position by the mechanical locking system based on a bias spring.

### 4.1. Aerodynamic and Operative Requirements

The selected case study involves a segment-B vehicle equipped with a front air dam. This component, typically in the form of a vertical plate on the front bumper, helps to separate the airflow at the rear of the car, significantly reducing the vehicle’s drag coefficient. Specifically, it decreases the overpressure area on the underbody mechanics. However, using such a component has some drawbacks, particularly at low speeds. To be effective, the air dam must be positioned very close to the ground, which increases the risk of it hitting the ground and breaking on steep or rugged roads. Therefore, it is advantageous to propose a movable geometry that replaces this component, providing aerodynamic performance comparable to the front air dam at high speeds, while eliminating the risks associated with low speeds by changing its configuration.

Therefore, CFD simulations have been carried out to define the geometries of the above-mentioned configurations. The numerical method consists of steady RANS, uses k-ε for turbulence modeling, and considers an inlet velocity of 140 kph to simulate the behavior of the vehicle at high speeds.

**Figure 4:**
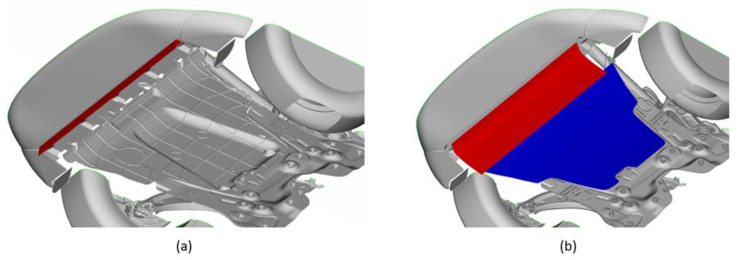
Configurations. (**a**) Base model with air dam; (**b**) skid plate modification with mobile part (in red) and fixed part (in blue).

The optimal shape has been achieved by eliminating the front air dam (Figure 4a) and assuming that a part of the skid plate is mobile (Figure 4b). This mobile part in the non-actuated configuration reproduces the aerodynamic performances of the front air dam.

**Figure 5:**
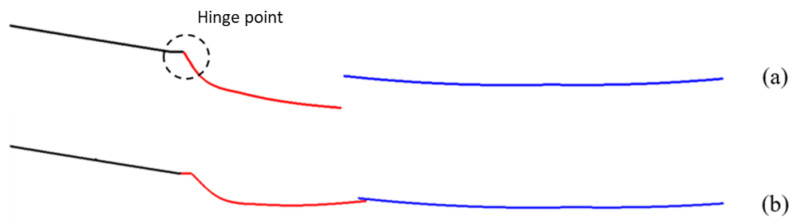
Skid plate configurations. (**a**) Deactivated configuration; (**b**) actuated configuration.

The skid plate has been rotated around the hypothesized hinge point, obtaining the two configurations shown in Figure 5. The red line represents the section of the movable part, while the blue one represents the fixed component.

The three configurations were then analyzed to verify the aerodynamic performances. The aerodynamic results are reported in Table 1.

**Figure 6:**
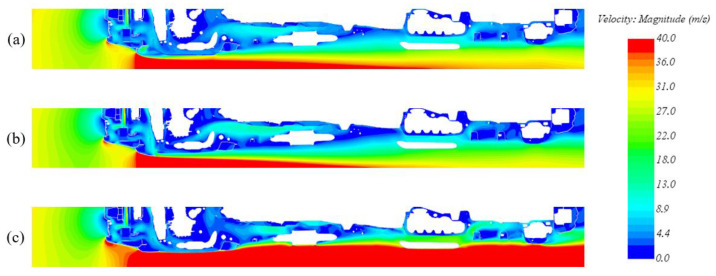
Velocity magnitude contour for the three configurations. (**a**) Base model; (**b**) deactivated configuration; (**c**) actuated configuration.

Two considerations can be made from the obtained results:

- The aerodynamic behavior of the deactivated configuration is comparable with the base model. The front air dam removal and the new geometry of the skid plate have no significant impact either on the total C_x_ value of the car or on the velocity field, which is very similar to the base model one.
- The aerodynamic behavior of the activated configuration shows the necessity and the purpose of this activity. It is not possible to simply consider the removal of the front air dam, as this would have a significant impact on the vehicle total C_x_ with an increase of about 7%, justified by the velocity field shown in Figure 6 (without the effect of the front air dam, the flow impacts directly on all the components in the underhood, significantly increasing the pressure on the underbody mechanical components).

To briefly summarize, at low speeds, the aerodynamic behavior of the activated solution does not differ from the basic model, but it has the advantage of removing the fixed component to avoid frequent failures, given its proximity to the ground. At low speeds, on the other hand, the C_x_ coefficient is higher; however, due to the decreased speed, the aerodynamic force acting on the skid plate is significantly reduced, thus making the substitution of a fixed skid plate with a mobile one a beneficial option.

### 4.2. Skid Plate Geometrical, Material, and Boundary Conditions Description

**Figure 7:**
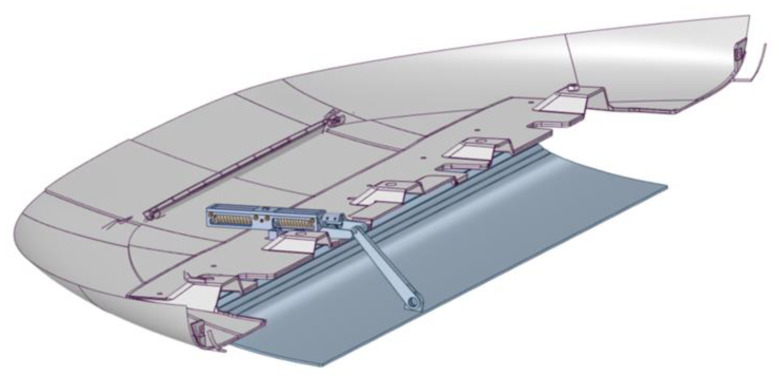
Skid plate system.

A hinge system is used to connect the actuator to the skid plate and convert the finite linear displacement of the actuator into the rotation of the plate. The assembled system is shown in Figure 7.

This system has two stable configurations (actuated and deactivated), and no external energy is required to keep the device in one of the other stable positions.

The actuator is required to guarantee a cover rotation of 12°, i.e., an upward displacement of 42.9 mm.

As a preliminary analysis, it has been verified in the CAD software that the 5 mm linear displacement of the actuator is enough for the rotation of the skid plate.

**Figure 8:**
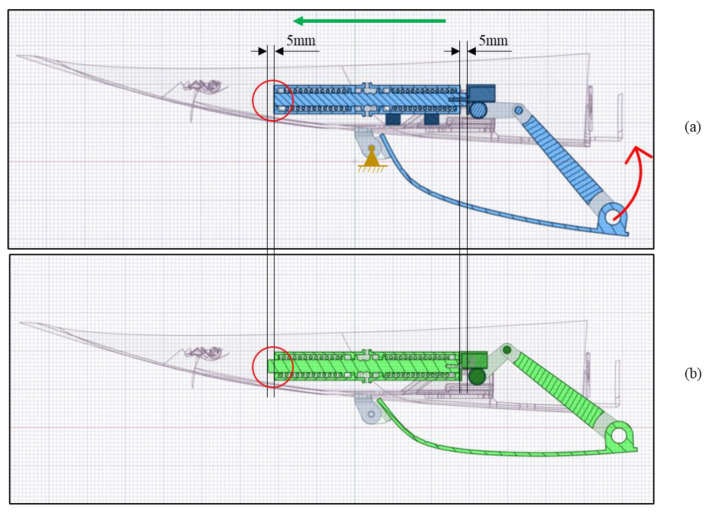
Linear actuator displacement. (**a**) First configuration; (**b**) second configuration.

In Figure 8, the kinematic motion is shown in a section view to better appreciate the details of the mechanism. To rotate the gear wheel attached to the cover, the actuator’s central body moves 5 mm toward the left (green arrow).

**Figure 9:**
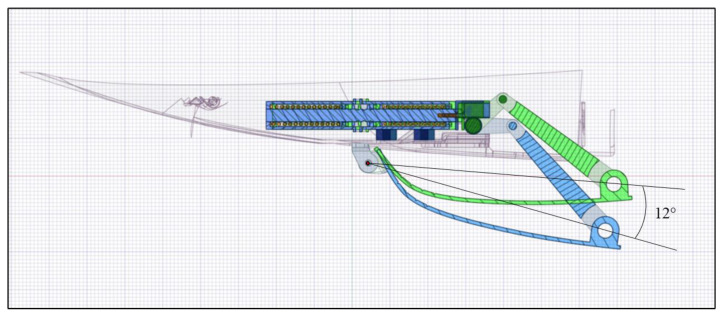
A 12° rotation around the fixed hinge.

The actuator’s linear motion results in a 12° rotation (red arrow) around the hinge (orange in the figure), thereby enabling the cover to be elevated by approximately 42 mm in relation to the vertical axis (Figure 9).

The individual components of the SMA-actuated skid plate are described hereafter in detail.

#### 4.2.1. Skid Plate

**Figure 10:**
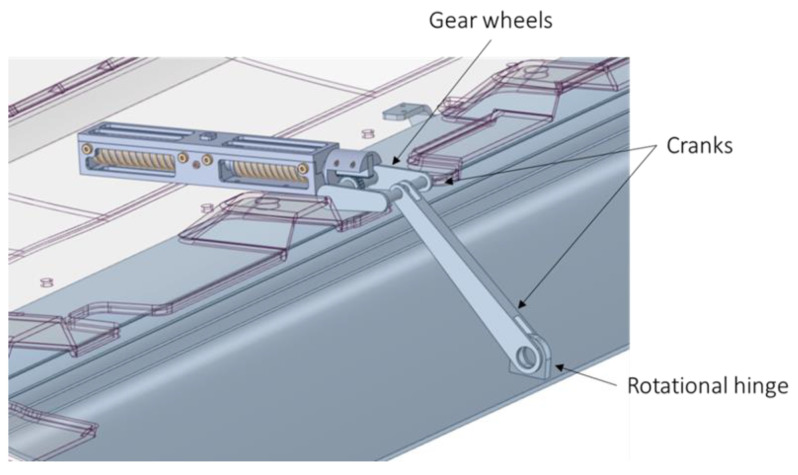
Identification of all components of the actuation mechanism.

The system consists of gear wheels, cranks, and hinges that allow the bottom plate to be rotated (Figure 10).

**Figure 11:**
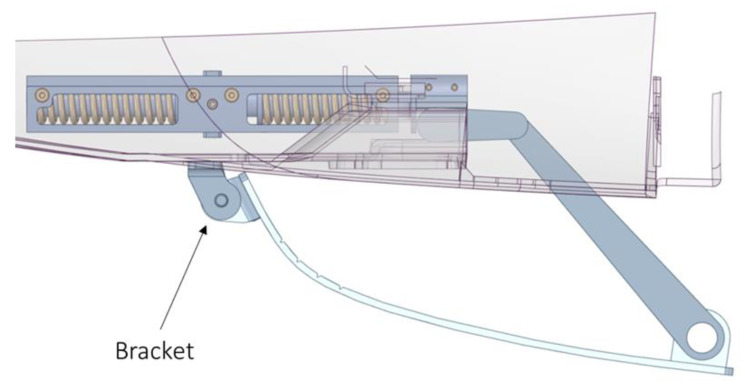
Brackets at the location of the hinge, which connects the cover to the rest of the car.

The linear movement induced by the actuator rotates the gear wheel mechanism. The latter moves the crank that moves the plate and rotates it around the center of rotation of the brackets (Figure 11).

**Figure 12:**
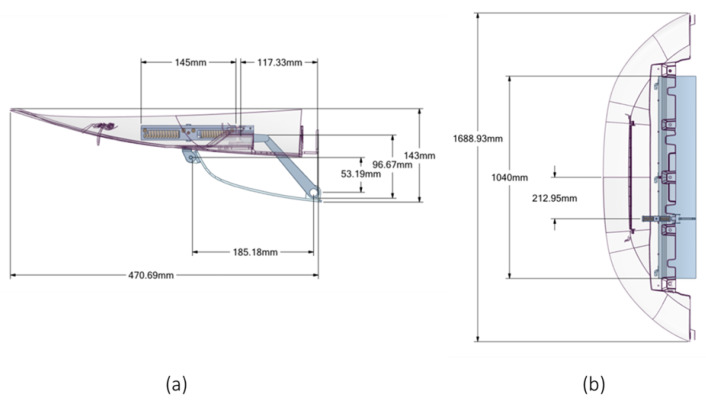
Geometrical description. (**a**) Lateral view; (**b**) top view.

In Figure 12, the main dimensions of the actuation system are shown.

#### 4.2.2. Bistable SMA Actuator

Bistable SMA actuators are much more efficient in comparison to other actuators, as can be seen in Table 2.

In terms of the forces exerted, SMA actuators need low current values. Current values required to activate the springs range from 10 ampere to 30 ampere and influence the response time of the actuator because activation times range from 40 s to 3 s. Having a bistable actuator means that the SMA springs do not deteriorate. In fact, once actuation has taken place, the current is removed, and the actuator remains stationary in the second equilibrium position.

**Figure 13:**
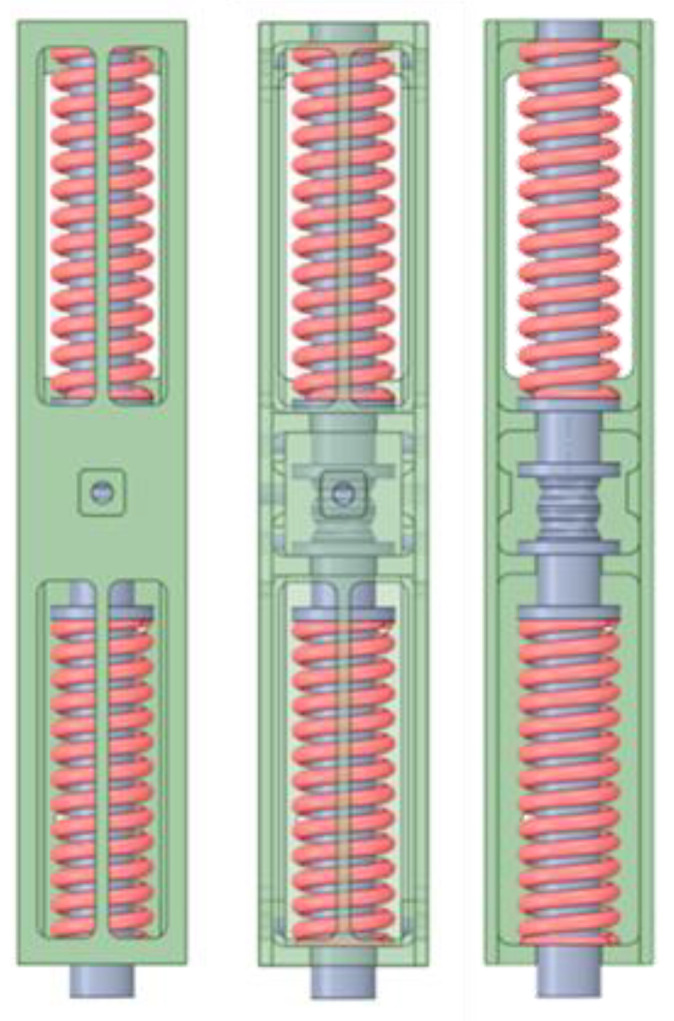
Detailed view of the SMA actuator.

The bistable actuator, previously introduced, consists of a central body that provides a guide for the springs and establishes the maximum explicable stroke, the two SMA springs that operate in phase opposition, and two lateral springs that represent the locking system. The actuator assembly including the outer case is shown in Figure 13.

**Figure 14:**
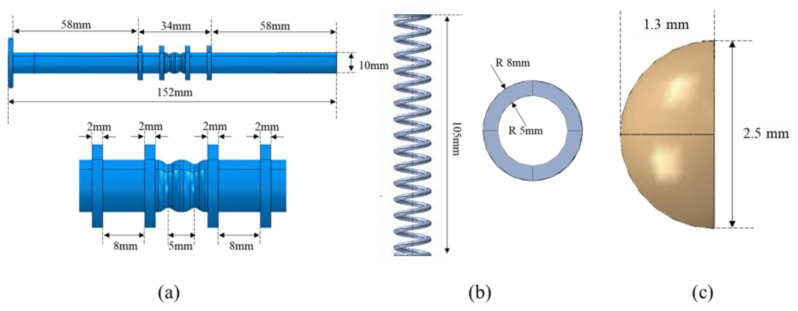
(**a**) Central body; (**b**) SMA spring; (**c**) locking system.

All actuator geometric properties are shown in Figure 14. The central body of the actuator has two antibuckling guides that ensure proper compression and expansion of the springs along a given axis. The total stroke of the actuator is 5 mm, and the two equilibrium positions can be seen in Figure 14a. The two SMA springs have the same dimensions and are shown in Figure 14b. The locking system consists of two hemispheres, which are shown in Figure 14c.

**Figure 15:**
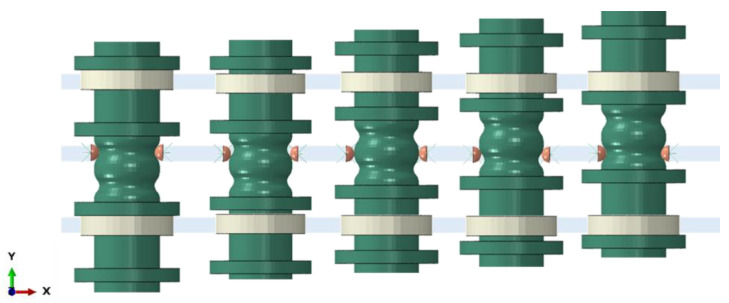
Locking system description.

The locking system consists of two metal hemispheres connected to two traditional metal springs (Figure 14c). These springs have a stiffness of 14 N/mm, allowing the actuator to lock and release. Figure 15 shows a front view of the operation of the locking system to highlight the movement of the hemispheres in the x-y plane. Activation of the SMA springs requires the central body to move along the y-axis. When the SMA springs are activated, the translation and special shape of the central body cause the hemispheres to be pushed outward against the action of the metal spring until they reach their new position. The metal springs then push and lock the hemispheres into the second horse saddle-shaped area until the other SMA spring is activated.

The operation of the entire actuator is based on the transformation of the SMA springs from the austenite to the martensite phase and vice versa.

The thermomechanical properties of the adopted materials, NiTiNOL (SMA springs), steel (central body, hemispheres, and hinges), and ABS (skid plate) are, respectively, shown in Table 3, Table 4 and Table 5. 

**Figure 16:**
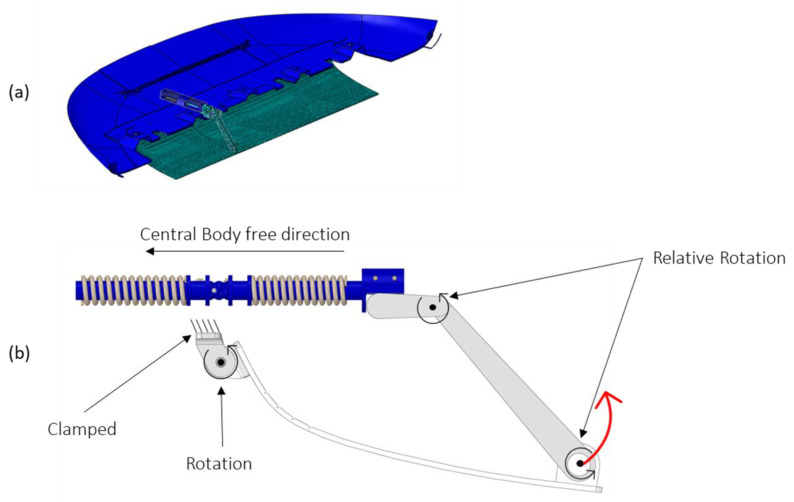
(**a**) FE model (skid plate is a “display body” with no mesh assigned); (**b**) boundary conditions.

An image of the FEM model and the applied boundary conditions are described in detail in Figure 16. The lower cover of the underbody shield is attached to the car’s frame and is allowed to rotate on a 12° axis. By heating one of the two SMA springs (the one on the right), the actuator moves toward the left and rotates the connection with the cover through the gear wheel (red arrow). The connecting rod is only constrained to rotate around the two axes indicated in the figure.

Moreover, an applied displacement preload of 55 mm and a constant temperature of 100 °C has been applied to the SMA springs to allow the complete transition to the austenitic phase.

### 4.3. Results and Discussion

The objective of this feasibility study was to conduct a preliminary exploration of actuator designs and performance metrics.

In this section, the numerical results of the work conducted are shown. The simulations of the SMA-actuated skid plate thermomechanical behavior can be subdivided into three stages. The first step concerns the force computation required to move the mechanism. 

A preliminary analysis has been performed on the mechanism without the use of the SMA springs and, therefore, without UMAT. The analysis has been conducted by imposing a displacement of 5mm, which is the value of the actuator stroke. In this way, the force required to move the skid plate mechanism has been computed. 

**Figure 17:**
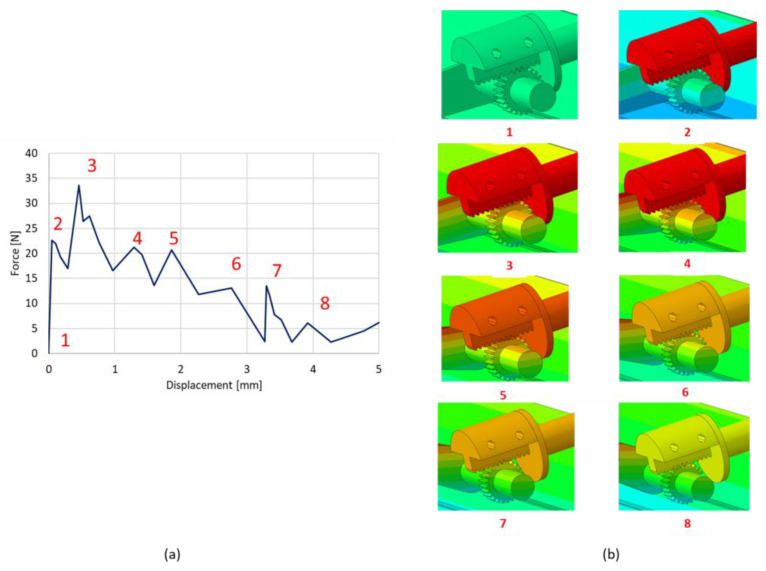
The force required to move the entire mechanism and deformations of the various step movements. (**a**) Force—Displacement graph; (**b**) Gear wheel rotation mechanism observed at different time instances.

As can be seen in Figure 17, the force required for the motion has several peaks, with the maximum value being 35 N. These peaks result from the gearwheel rotation mechanism, which can be seen in the detail on the right.

**Figure 18:**
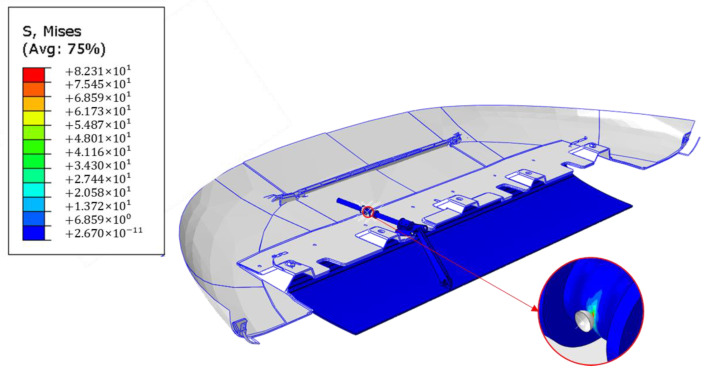
Von Mises distribution.

This analysis has been performed on the model without the introduction of the SMA spring in order to evaluate the load transmitted by the cranks. In Figure 18, the contour plot of stress distribution on the deformed shapes of the skid plate is shown. The maximum stress value is on the actuator axis at the lateral spring transition.

The second stage has been focused on the evaluation of the maximum force developed by the SMA springs at the martensite–austenite phase transition.

To ensure the required force, a customized sizing of the shape memory alloy spring’s geometry was conducted. Dynamic effects were left out of the simulation since they are assumed to have negligible influence on the global behavior of the actuator. 

The analysis has been performed on the SMA spring fully constrained at an edge. A first mechanical load has been applied to the SMA spring in terms of applied displacement (55 mm) in order to bring the spring to the $\sigma_{Mf}$ stress value to allow the transformation in the martensite phase. Then, a constant temperature of 373 K has been applied to the SMA springs in order to allow the transition to the austenite phase.

**Figure 19:**
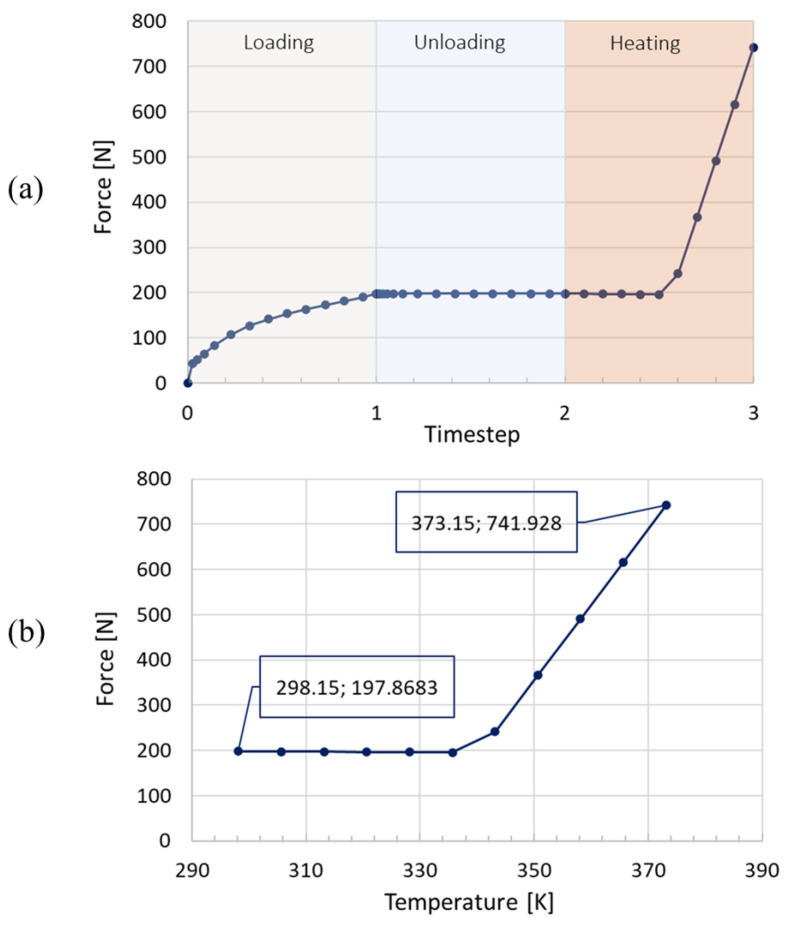
SMA Spring. (**a**) Force—timestep; (**b**) force—temperature.

Figure 19 clearly shows the starting temperature (298.15 K) and the heating temperature (373.15 K) to recover part of the deformation. Moreover, the maximum SMA spring force is 740 N, which is higher than the locking spring-induced force. In addition, Figure 19 illustrates a single shape recovery cycle since the task, at this stage of the design and analysis process, has been to ensure the successful implementation of the bistable actuator using shape memory alloys.

**Figure 20:**
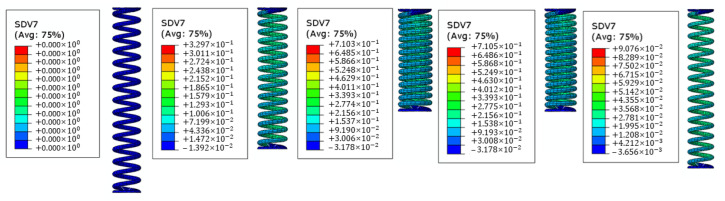
Evolution of the martensite volume fraction in the SMA spring during the phase transformation.

In Figure 20, the evolution of the volume fraction of martensite in the SMA spring (variable SDV7) is shown for four different timesteps of the analysis (for a spring that is unloaded and cold, for a spring hat is half preloaded and cold, for a spring that is fully preloaded and cold, for a spring that is unloaded, and for a hot spring). These results are a further confirmation of the accurate prediction provided by the UMAT SMA routine.

Figure 20 shows that the transformation to austenite is not fully completed, which enables an actuator to release from its first stable position with less energy input. Moreover, it highlights that the force responses of the SMA springs can be significantly improved by optimizing their geometry.

An experimental study has been performed on the SMA springs to assess their actuation force. Specifically, the designed spring has been subjected to a test campaign to determine the active point, which represents the optimal point of actuation. The actuation system allows the net force to be evaluated.

**Figure 21:**
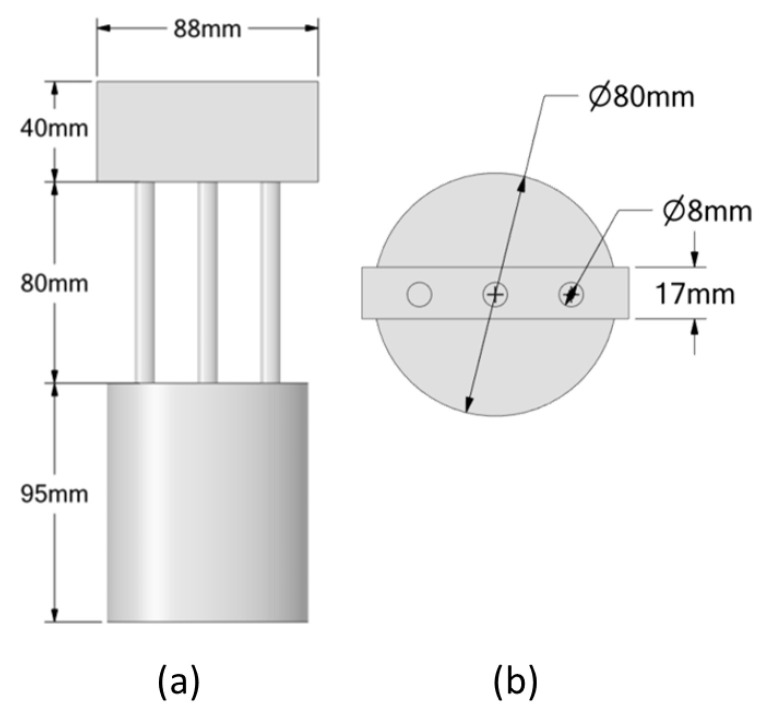
SMA bias fixture dimensions. (**a**) Frontal view; (**b**) top view.

The fixture designed for testing is depicted in the Figure 21. The implementation requirements must ensure that during the heating phase, the SMA spring performs a $\frac{F}{F_{actuation}}\geq 10$.

Tests were carried out using a universal testing machine equipped with a 5 kN load cell and an 800 mm stroke at a crossbeam speed of 0.2 mm/s. Three different types of K thermocouples are positioned on the top, middle, and bottom parts of the SMA spring to control the temperature.

**Figure 22:**
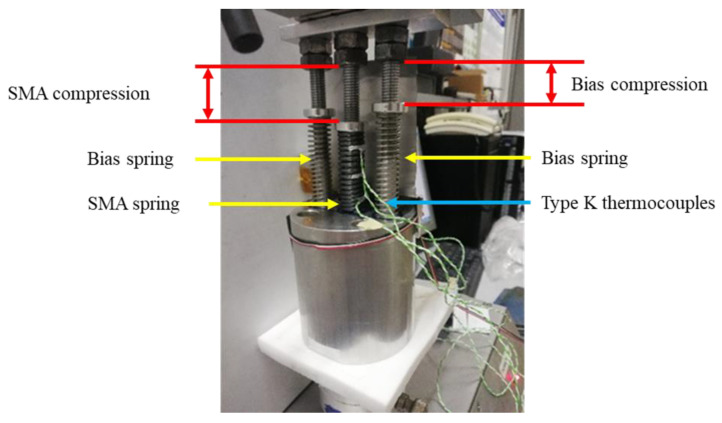
Experimental setup.

Figure 22 displays the experimental set of springs utilized for the active point evaluation. The design of the fixture permits the accommodation of two bias springs and one SMA spring, with the SMA spring positioned between the two bias springs to minimize the moments resulting from its actuation. The bias springs and the SMA spring positioned inside the fixture are represented by yellow arrows, while the three K-type thermocouples are identified by a blue arrow. The compressions of the bias springs and the SMA springs are indicated in red.

To evaluate the performance of the SMA actuation device as a function of increasing temperature, various combinations of the SMA and bias springs, as well as active points, are examined. The SMA spring temperature has been recorded by thermocouples. In addition to these, a fourth external thermocouple, placed approximately 1 cm from the spring, has been inserted to measure the ambient temperature during the test. This measurement makes it possible to quantify the shape memory effect in the SMA. The force values measured and shown in the table represent the force produced by the SMA spring net of the two bias springs.

The evaluation of the SMA force involved examining three different configurations. The SMA spring has been subjected to varying displacements of 55 mm, 59 mm, and 64 mm, while a compression of 45 mm was computed for the bias spring. The tests are performed for four load and unload cycles. Table 6 presents the findings of the experiment, including the maximum values observed by both the thermocouples and the load cell. After testing all the configurations, it has been determined that they all meet the required force specification. However, the first configuration stands out as the most favorable due to its small footprint, making it the most space-efficient option.

**Figure 23:**
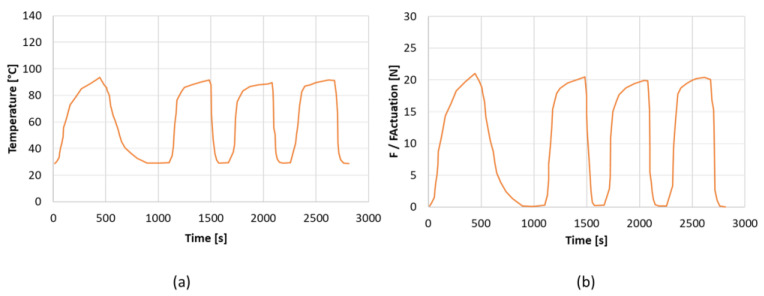
Experimental results. (**a**) Load history; (**b**) temperature history.

In Figure 23, the load and temperature histories of the best configuration is shown. 

In the third stage, a comprehensive verification of the SMA-actuated skid plate has been conducted. A full simulation with all the components and the complete set of boundary conditions and loading conditions has been performed in seven steps. First, a preload in the SMA spring has been applied up to the full transition to the martensite phase. In these conditions, the skid plate system is kept blocked by the locking mechanisms. Then, the two side springs arranged radial to the actuator axis by 180° have been precompressed by a displacement of 0.529 mm. Once the displacement of the lateral springs has been applied, a step has been created in which the release of the lateral springs takes place.

The fourth step concerns the heating phase. A heating temperature of 373 K has been considered. A lower temperature could not be considered because it would allow the springs to be activated even when not necessary by the temperature reached in the car in operative scenarios. To evaluate the entire mechanism, the functioning of both springs has been validated. Hence, the heated spring is cooled down, followed by a stabilization step of the entire mechanism. Finally, the second spring is heated.

**Figure 24:**
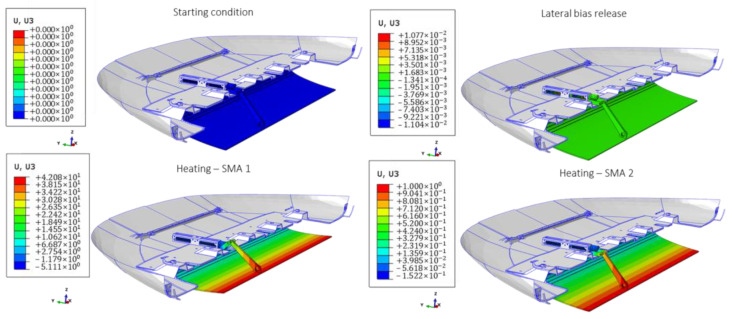
SMA-actuated skid plate full simulation. Displacement distribution.

In Figure 24, the main four configurations of the SMA-actuated skid plate resulting from the seven steps are introduced. It can be observed that the actuator is able to guarantee the necessary displacement of the skid plate during the heating phase of the first SMA spring. Additionally, it is capable of reverting to its initial configuration after the thermal load has been applied to the second spring.

**Figure 25:**
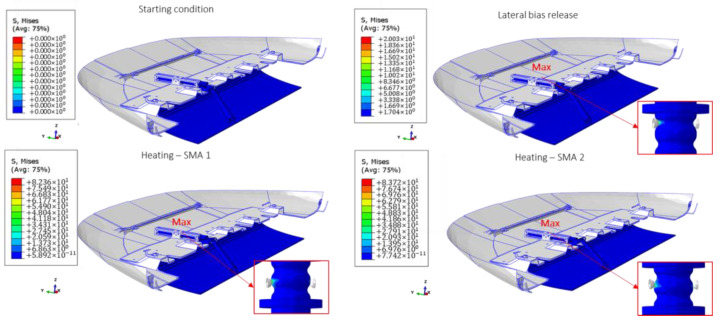
SMA-actuated skid plate full simulation. Von Mises distribution.

In Figure 25, the main four configurations of the SMA-actuated skid plate resulting from the seven steps are introduced. The maximum value is computed on the central body of the actuator throughout the entire FEM analysis.

Based on the results of the preliminary analyses, it is evident that the designed SMA bistable actuator can rotate the skid plate system 12° around the fixed hinge, resulting in a 42 mm displacement at the end in the upward and downward vertical directions. According to experimental validation of the SMA springs and actuator dimensioning [54,55], the main properties of the SMA springs for the implementation of the complete numerical model have been derived. In conclusion, the force exerted by the SMA springs is 21% greater than the force required by the system. This is an indication of how the actuator can still be optimized by working on the volumetric fraction of martensite, which is indicative of material transformation. However, at the same time, it shows how it can be used for various engineering applications and investigations.

## 5. Conclusions

The following conclusions can be drawn from the results of this study on the design and analysis of a bistable SMA actuator for the movement of a skid plate system. The scope was to develop a low-complexity and low-energy-consumption bistable actuator utilizing the characteristic properties of shape memory alloys.

- Based on the aerodynamic study, rotating the plate by 12° in two stable configurations led to a significant improvement in its durability, but did not significantly enhance its aerodynamic performance.
- A preliminary analysis to verify the kinematic motion revealed that a force of approximately 35 N is required to move the skid plate system.
- Using the UMAT in Abaqus software, the thermomechanical properties of both shape memory alloy effects were simulated. The numerical analysis results indicate that the SMA spring system produces a maximum force that is 21% greater than the required force.
- To verify the force generated by the SMA spring, three experimental tests were performed. Although all tested configurations met the force requirement, the first configuration, which has the smallest footprint, was selected.
- A final analysis of the entire system demonstrated the feasibility of the proposed actuator and the stability of the two possible configurations of the SMA skid plate system.

## Figures and Tables

**Table 1:** C_x_ results of the analyzed configurations.

	C_x_	Delta C_x_ (%)
Base model	0.309	-
Deactivated configuration	0.310	+0.3
Actuated configuration	0.332	+7.4

**Table 2:** Comparison of actuator performance [31].

Actuator Type	Work per Volume (J/cm^3^)	Power per Volume (W/cm^3^)
Hydraulic	5	20
Pneumatic	0.175	3.5
NiTi SMA	10	30

**Table 3:** NiTiNOL thermomechanical properties [54,55].

Nitinol			
$\sigma_{scr}\left[MPa\right]$	15	$\alpha_{M}\left[1/K\right]$	$2.2\times 10^{-6}$
$\sigma_{fcr}\left[MPa\right]$	100	$\alpha_{A}\left[1/K\right]$	$2.2\times 10^{-6}$
$E_{M}\left[GPa\right]$	51.87	$\varepsilon_{l}$	0.05
$E_{A}\left[GPa\right]$	115.87	$M_{s}\left[K\right]$	338
$C_{M}\left[MPa/K\right]$	8	$M_{f}\left[K\right]$	303
$C_{A}\left[MPa/K\right]$	8	$A_{s}\left[K\right]$	328
$\nu_{M}$	0.33	$A_{f}\left[K\right]$	381
$\nu_{A}$	0.33		

**Table 4:** Steel mechanical properties.

Steel	
E [GPa]	210
ν	0.33

**Table 5:** ABS mechanical properties.

ABS	
E [GPa]	2.6
ν	0.33

**Table 6:** Experimental results.

Configuration	SMA	BIAS	Tmax	$F/F_{actuation}$
#1	55 mm	45 mm	93	21
#2	59 mm	45 mm	86	19
#3	64 mm	45 mm	90	18

## Data Availability

The processed data required to reproduce these results are available throughout the manuscript.
